# Agency, Structure and the Power of Global Health Networks 

**DOI:** 10.15171/ijhpm.2018.71

**Published:** 2018-08-04

**Authors:** Jeremy Shiffman

**Affiliations:** ^1^Johns Hopkins University, Bloomberg School of Public Health, Baltimore, MD, USA.; ^2^Paul H. Nitze School of Advanced International Studies, Washington, DC, USA.

**Keywords:** Global Health Policy, Global Health Networks, Global Health Governance, Constructivism

## Abstract

Global health networks—webs of individuals and organizations linked by a shared concern for a particular condition—have proliferated over the past quarter century. In a recent editorial in this journal, I presented evidence that their effectiveness in addressing four challenges—problem definition, positioning, coalitionbuilding and governance—shapes their ability to influence policy. The editorial prompted five thoughtful commentaries that reflected on these and other challenges. In this follow-up editorial, I build on the commentaries to suggest ways of advancing research on global health networks. I argue that investigators would do well to consider three social theory-influenced global governance debates pertaining to agency—the capacity of individuals and organizations to act autonomously amidst structural constraints. The three debates concern the relationship between agency and structure, the power of ideas vis-à-vis interests and material capabilities, and the level of influence of non-state actors in a global governance system that most scholars identify as state-dominated. Drawing on these debates, I argue that rather than presume global health network influence, we need to find more robust ways to investigate their effects. I argue also that rather than juxtapose agency and structure, ideas and interests and non-state and state power, it would be more productive to consider the ways in which these elements are intertwined.

## Introduction

Over the past quarter century global health networks—webs of individuals and organizations linked by a shared concern for a particular condition—have proliferated. In a recent editorial in this journal,^1^ drawing on case studies of eight global health networks^ [1] ^, I identified four challenges that these networks commonly face. I presented evidence that network effectiveness in addressing these challenges shapes their capacity to influence policy. The editorial prompted five thoughtful commentaries that reflected on these four challenges, on other factors potentially influencing global health networks, and on strategies to augment network effectiveness.^2-6^ The challenges are:

1. *Problem definition*: generating internal consensus on the nature of the problem and solutions
2. *Positioning*: portraying the issue in ways that inspire external audiences to act
3. *Coalition-building*: forging alliances with actors beyond those in the health sector
4. *Governance*: establishing suitable institutions to facilitate collective action.

In this follow-up editorial, I build on the insights of the commentaries, and connect the commentaries and original editorial to three social theory-influenced global governance debates concerning agency—the capacity of individuals and organizations to act autonomously amidst structural constraints. Constructivists—scholars who understand much of reality to be socially constructed—have played a large role in bringing these debates into the scholarly field of global governance. The three debates pertain to the relationship between agency and structure, the power of ideas vis-à-vis interests and material capabilities, and the level of influence of non-state actors in a global governance system that most scholars identify as state-dominated. The commentaries and my original editorial present explicit evidence or make implicit assumptions affirming the power of agency, ideas and non-state actors.

I argue that scholars investigating global health networks would do well to reference these debates as a means of advancing inquiry on global health network effects and contributing to theory development on the nature of agency in global governance. I make three primary points in this regard. First, rather than presume global health network influence, we ought to find more robust ways to investigate whether they have significant effects on policy and population health, and if so under what circumstances. Second, global health network initiatives are examples of a larger phenomenon—attempts by transnationally-linked actors to induce normative change in the global system—and their study potentially can contribute to theory development on this subject. Third, as we investigate global health networks and their effects, rather than juxtaposing structure and agency, interests and ideas, and state and non-state power, it would be more productive to consider the ways in which these elements are intertwined.

## Structure and Agency

At the heart of the structure-agency debate in global governance is the extent to which individuals and organizations can alter the world in the face of durable social arrangements that may constrain change (that is, structural barriers). Scholars working in a constructivist tradition^7-11^ have drawn on social theory^12-15^ to inject structure-agency debates into the global governance field.

Constructivists disagree on the power of agency.^16^ Sociological institutionalists emphasize structural influence and downplay agency.^9^ They note the remarkable similarity in the composition and aspirations of states that, they argue, cannot be explained without appeal to the power of a world culture, one that privileges rights and modernity. This world culture is a homogenizing force, one that constrains agents who are engaged less in careful deliberation than in the somewhat mindless enactment of received scripts. To illustrate this power of a world culture, Meyer and colleagues imagine that (pp. 145-6)^9^ :

“If an unknown society were “discovered” on a previously unknown island, it is clear that many changes would occur. A government would soon form, looking something like a modern state…. Official recognition by other states and admission to the United Nations would ensue.... Its people would be formally reorganized as citizens with many familiar rights…. Standard forms of discrimination, especially ethnic and gender based, would be discovered and decried. The population would be counted and classified in ways specified by world census models.”

Agentic-oriented constructivists, while recognizing the power of structure, criticize the mechanistic thrust of sociological institutionalism, seeing contingency in the world and the role of human consciousness and deliberation in change. For instance, Finnemore and Sikkink^10^ point to the example of Henry Dunant, who inspired the formation of the International Committee of the Red Cross in 1863 and helped to alter the rules of warfare to include, among other norms, the neutrality of non-combatants. Another example they mention are women’s rights advocates who in 1904 established the International Women’s Suffrage Association, marking the launch of an international campaign that resulted in the widespread adoption of voting rights for women. Sikkink,^11^ concerned about what she understands to be the dominance of structural perspectives in constructivism, has called for an ‘agentic constructivism,’ one that is (p. 8):

“…concerned with the micro-foundations of creating and constituting new actors and new conditions of possibilities—where new actors take on and challenge (and sometimes change) existing logics of appropriateness…. These actors don’t mindlessly ‘enact’ or ‘perform’ scripts, but question them.”

Most individuals and organizations that comprise global health networks, presumably, believe in the power of agency, expecting through their work to reduce levels of morbidity and mortality that arise from the conditions that concern them. A challenge to this perspective is the idea that global health networks are epiphenomenal—a product of the existence of health conditions but having no sizeable impact on their severity or on global and national efforts to address them. A variant of this argument is that their members are not exercising agency so much as enacting scripts produced by the homogenizing, modernistic world culture that sociological institutionalists claim exists.

The original editorial and commentaries, although cognizant of structural influence, recognize the power of agency and seem to reject a position that structure is overwhelmingly constraining and that networks are epiphenomenal (italics added):

“Problem and solution definition and external positioning…can have *an important influence on*…*public health programs”* (p. 2).^4^

“The way networks manage these four challenges *has substantial influence* on the likelihood that they *achieve their objectives”* (p. 188).^1^

“[The NCD Alliance] is a classic example of *a successful network*, bringing coherence and strength to a formerly diffuse community” (p. 3).^2^

Empirical research has yet to settle the question of the epiphenomenal nature of networks or the size of network effects, however. Moreover, detecting network effects is a difficult research challenge, as colleagues and I discovered as we undertook case studies of networks addressing alcohol harm, tobacco control, tuberculosis, pneumonia, maternal survival, newborn survival, surgical conditions and early childhood development.^17-24^ The further one moves down the policy funnel—from global resolutions to national uptake to population health—the more difficult it becomes to assess network effects, since the number of confounding factors multiplies.^25^

While many network members and published reports make strong assertions about network efficacy, social scientists must stand back from these claims and find robust means of assessing effects. One strategy is to consider counterfactuals: in the absence of a particular network, would attention to and the prevalence of a condition be significantly different? We can never know for certain, since we cannot rerun history and compare outcomes in worlds with and without that network. But we can imagine what the world would be like without a particular network and make cautious inferences on network effects on that basis. Beyond this, we might set up matched comparisons that allow us to gain some control over alternative explanations for policy and population health change. Specifically, we could compare networks matched as much as possible on issue characteristics (such as the nature of the vector, the ease of transmissibility of the condition and its prevalence in high-income countries) and elements of the global political context (for instance, whether the condition has a dedicated Sustainable Development Goal target—although we would need to account for the possibility that the target itself is a product of network agency). Doing so offers leverage to assess whether differences in policy and population health change across conditions may be due to network activity^ [2] ^. Another strategy is to consider hard cases where we would not expect extensive network influence due to political sensitivities or other reasons, such as efforts to liberalize national abortion laws. In all these research strategies, investigators are likely to benefit by using process-tracing methodologies to detect mechanisms of influence.^26^

As they investigate global health network effects, researchers might benefit by drawing on social theory to consider how agency and structure are linked. For instance, Giddens’ theory of structuration^12^ recognizes the mutual constitution of agency and structure: agents reproduce structure as they act. Sewell^15^ critiques and expands on that theory, identifying both ideational and material elements to structure, and the power of agency to alter structure. Bourdieu^14,27^ draws on the concepts of agency and structure to develop a theory of social worlds—such as law and medicine—organized as fields, constituted by actors with shared dispositions but unequal positions who deploy various forms of capital to advance their interests. Fligstein and McAdam^28^ expand on Bourdieu’s theory of fields to consider not just their stability but also how, via individual and collective agency, fields change. Wendt^7^ brings social theory into the international relations and global governance fields, arguing for theories grounded in the mutual constitution of structure and agency—rather than ones that have an exclusively structural or individualistic ontological foundation. All these theorists offer ways of considering how global health networks as agents potentially change the world, but do so constrained (and facilitated) by social structures and reproducing (and altering) these structures as they act.

## Interests and Ideas

Global governance scholars debate not only the power of agency, but also the substance of that power. Most scholars working in rationalist and Marxist traditions^29-31^ view power as serving interests, derived from control over material resources such as financing, the means of production and coercive apparatuses. Constructivists point to another form of power: ideas, among which are norms—principled beliefs concerning appropriate behavior for actors with a given identity^32^ —and knowledge^ [3] ^. ^33^ They argue that actors in global governance operate not just from a logic of consequences—self-interested calculations surrounding means and ends—but also from a logic of appropriateness—a sense of right and wrong.^34^ The cross-national spread of women’s suffrage and abolition of slavery are two examples of the power of norms.^10^

If global health networks influence policy and population health, do they do so largely through ideational power, material power or an amalgam? Are they motivated by principled beliefs, interests, or some combination? While recognizing material forces and interests, the original editorial and many of the commentaries, consistent with constructivism, ascribe power to ideas:

“Research in social psychology and social movement scholarship has found that alignment in framing is important for persuasion and the adoption of new policies” (p. 2).^4^

“The power of the social construction of issue framing” (p. 1).^6^

Some of the commentaries also point to the importance of material resources:

“While catalytic funding for some health alliances has been forthcoming…there has been no equivalent for other NCD-relevant areas such as physical activity or alcohol” (p. 3)^ [4] ^. ^2^

Like the power of agency, the extent of influence of ideas vis-à-vis material forces on global governance outcomes is far from settled. One promising avenue for advancing inquiry on this subject is an integrative research agenda in global governance that challenges the ideational-material and norms-interests divides. Sil and Katzenstein,^35^ for instance, call for abandoning paradigm-bound research that focuses either on interests or norms, in favor of considering multiple perspectives— ‘analytical eclecticism’ in the study of international relations. Sell and Prakash^36^ point out that aside from profit, businesses also pursue normative concerns, and that non-governmental organizations ((NGOs), presumed to be motivated by principle, are concerned with their material well-being. Mitchell and Schmitz,^37^ examining transnational NGOs, link norms and resource-maximizing behavior through the concept of ‘principled instrumentalism.’ Robinson provides evidence that both ideational forces—the normative influence of NGOs—and material forces—a country’s indebtedness to the World Bank—are associated with a wave of national adoption of population policies in Sub-Saharan Africa in the 1980s and 1990s.^38^ Finnemore and Sikkink,^10^ although recognized as constructivists, reject the norms-interests division as simplistic, advancing the idea that much social change can be understood via processes of strategic social construction—actors instrumentally pursuing principled concerns to alter social reality. They make a call to scholars: “Instead of opposing instrumental rationality and social construction we need to find some way to link those processes theoretically” (p. 910).

Research on global health networks might benefit from attending to this call. Members of global health networks may be motivated simultaneously by multiple imperatives^10^ : commitment to principled ideas, altruism, the search for knowledge, empathy for individuals at risk, the pursuit of self-esteem, the desire for the respect of their colleagues, the survival of their organizations, the acquisition of authority in the field of global health, the undoing of rivals, financial gain and geopolitical interest. And their power may be both ideational—a function of the potency of their principled ideas and epistemic claims—and material—a result of their control over financial and other kinds of resources. This complexity suggests the value of an interdisciplinary research agenda to detect the many potential sources of global health network motivation and power, drawing on insights from, among other fields, social psychology, organizational behavior, economics, sociology, anthropology, history, public administration and political science. This complexity also suggests the value of drawing on the work of the social theorists mentioned above to investigate the mutual constitution of structure and agency, and specifically how agents in global health networks construct their fields through their daily practices. Such investigations may lead us to challenge mono-causal accounts of actor motivation in favor of ones that consider how ideas of multiple forms come to constitute interests,^10^ and how ideational and material power are interwoven.^15^

### State and Non-state Actors

A third global governance debate of relevance to the study of global health networks pertains to how much power non-state actors have in a global system that most analysts consider to be state-dominated. Keck and Sikkink^39^ highlight the power to alter state behavior of transnational advocacy networks (TANs)—cross-nationally linked networks of mostly non-state actors pursuing principled concerns such as human rights. Haas^33^ points to the power of epistemic communities—networks of experts in a particular field such as climate change—to do the same^ [5] ^. They and other constructivist-oriented scholars have challenged state-centric paradigms—particularly neorealism—that take the material capabilities of states, in pursuit of self-interested concerns such as security, as the only influence on outcomes in the international system worth attending to^ [6] ^.

Several of the commentaries, interpreting global health networks to be non-state actors, highlight their power. Marten and Smith,^3^ in a counterpoint commentary on the original editorial, argue that states are a prerequisite for global health network effectiveness and that studies in the field of global health governance have overlooked the role of state power. Their points are well-taken, particularly in emphasizing the ongoing centrality of the state in the global governance system.

However, there are three difficulties with their argument^ [7] ^. First, they misconstrue global health networks to be exclusively non-state actors. Global health networks vary in composition. Some are comprised exclusively or predominantly of non-state actors, such as the Framework Convention Alliance for Tobacco Control from which they draw their primary examples, and the NCD Alliance, which commentator Dain leads. Many others are of mixed composition, linking state and non-state actors. For instance, the 1500 partners of the Stop TB Partnership include, “international and technical organizations, government programs, research and funding agencies, foundations, NGOs, civil society and community groups and the private sector.”^40^ In this regard it is worth going back to our original definition of global health networks (p. 2).^41^ State actors are encompassed in the definition (underlines added):

“Global health networks are cross-national webs of individuals and organizations linked by a shared concern to address a particular health problem global in scope. They may consist of and connect multiple types of institutions, including United Nations (UN) agencies, bilateral donors, international financial institutions, private philanthropic foundations, national governments, international and national NGOs, medical associations, research institutions and think tanks.”

Second, their use of the term ‘prerequisite’ for global health network effectiveness denotes a necessary condition, and therefore a very strong claim about state power. That claim should be investigated, not assumed, and we need to ask about effectiveness toward which end. It may be the case that state power is necessary for global health network effectiveness on nearly all outcomes of interest. However, it is more likely that the state’s role in global health network effectiveness varies depending on (1) the nature of the issue; (2) the strength and composition of the network; (3) the level of the system (ie, global, national, sub-national, community); and (4) the stage of the policy process (ie, agenda-setting, formulation, adoption, implementation, evaluation). In some circumstances, the proposition that state power is a prerequisite for global health network effectiveness seems plausible: for instance, national and sub-national policy implementation where state capacity is strong (in areas of limited statehood such as much of Somalia, the proposition may not always hold). In other circumstances, global health networks—even ones comprised predominantly of non-state actors—may have influence even in the initial absence of strong state concern: for instance, placing a previously overlooked but relatively uncontroversial issue, such as newborn survival, on global and national policy agendas. The point is that these are open empirical questions that require investigation.

Third, it is hard to sustain the claim they make that the thrust of global governance scholarship, and more specifically global health governance scholarship, has overlooked the role of the state—although a more nuanced claim that *some* works have underplayed the role of the state justifiably could be advanced. The opposite is more accurate: while there is certainly great and growing interest in non-state power, most global governance and global health governance scholarship has focused on the state, and most researchers working in these areas—appropriately in my view, and in line with Marten’s and Smith’s central concern—recognize states as the core and most powerful actors in the global governance system.

Rather than speak of prerequisites or of overlooking the state, there are more promising ways to frame this research agenda. One is to take up a valuable suggestion offered by Tosun^5^ to apply to global health the perspective of polycentric governance, associated with the work of Elinor Ostrom,^42^ often used in climate change research. Ostrom argued that (p. 552):

“Polycentric systems are characterized by multiple governing authorities at differing scales rather than a monocentric unit…Each unit within a polycentric system exercises considerable independence to make norms and rules within a specific domain (such as a family, a firm, a local government, a network of local governments, a state or province, a region, a national government, or an international regime)…. An important lesson is that simply recommending a single governance unit to solve global collective-action problems—because of global impacts—needs to be seriously rethought.”

Another idea for framing the research agenda, in line with the structure-agency and interest-norm global governance debates, is to ask about interconnections. In a global health governance system that most analysts appropriately recognize as centered on the state, how do states condition the power of non-state actors? How do non-state actors augment or challenge the power of the state? What role do state and non-state actors play jointly in network emergence? In short, in what ways are state and non-state power intertwined?

Global health networks offer rich empirical material to explore these questions. Under what circumstances are state interests the predominant influence on global health network objectives? Non-state interests? When do state and non-state preferences converge? How often do coalitions form linking state and non-state actors (potentially resulting in competing state-non-state alliances)? Under what circumstances are those global health networks comprised predominantly of non-state actors able to hold states to account? When are non-state actors the primary force in global health network formation? State actors? State-non-state alliances? Via process-tracing, historical case studies, matched comparisons, counterfactual analysis and other approaches, researchers might gain insights concerning the nature, power and effects of global health networks, and more broadly on the intersections of state and non-state power in the global governance system. And again, the social theorists noted above potentially provide valuable concepts for understanding these state-non-state interactions in the context of global health networks. They do so particularly by directing us to consider how historical processes and global social structures condition the very emergence of global health networks, and how these networks in turn, linking state and non-state power, refashion these same social structures: the ongoing mutual constitution of structure and agency.

## Conclusion

The five commentators raise thought-provoking questions about the power of global health networks in global health governance. Their remarks raise deeper issues pertaining to actor power, specifically surrounding the relationships between agency and structure, norms and interests and non-state and state power. Global governance scholars, particularly those working in a constructivist tradition, have debated these subjects. Some argue that the most promising way to advance these debates is to consider ways in which these influences are intertwined. I concur and suggest such an integrationist research agenda will benefit the study of global health networks and global health governance more broadly, while also potentially enabling global health research to contribute to theory development on the power of agency and normative change in the global governance system.

In conclusion I offer three ideas on advancing an integrationist research agenda on global health networks—one each pertaining to each of the three debates^43^ :

- *Mutual constitution of structure and agency:* Global health networks are products of historical conditions, but once created they alter the global health landscapes that they join. Global health networks likely reveal processes of mutual constitution of structure and agency, rather than the power of either structure or agency alone or in isolation. It would be valuable for researchers to be attentive to these processes of mutual constitution.
- *Complex motivations of global health actors:* The motivations of actors that comprise global health networks may be multiple, intertwined and unclear even to the actors themselves. In investigating these motivations, it may be worth setting aside, at least temporarily, the impulse to quickly label these as grounded either in ‘ideas’ or ‘interests’ and instead to direct efforts to detecting the multiple motivations that may be at work.
- *Fusion of state and non-state power:* Many global health networks blend state and non-state power. Rather than presume the primacy of either, we may gain greater insight by exploring just how such power is intertwined.

## Acknowledgments

JS would like to thank Yusra Shawar and Hans Peter Schmitz for their valuable comments on a draft of this editorial.

## Competing interests

Author declares that he has no competing interests.

## Author’s contribution

JS is the single author of the paper.

## Ethical issues

Not applicable.

## Endnotes


[1] Addressing alcohol harm, early childhood development, maternal survival, newborn survival, pneumonia, surgical conditions, tobacco control and tuberculosis.

[2] We employed this strategy in a project involving six of the eight case studies discussed in this editorial (see reference number 41). We compared networks addressing tuberculosis and pneumonia—two high-burden communicable diseases that predominantly affect the respiratory system; maternal and neonatal mortality—problems pertaining to two population groups at risk surrounding childbirth; and tobacco use and alcohol harm—two addictive substances. In each pair, despite comparable burden, the first has received greater global policy attention than the second. The project examined the role of networks in explaining this variance.

[3] Some scholars working in Marxist and rationalist traditions also recognize ideational power but view such power as advancing interests rather than principled beliefs. For instance, Gramsci argues that ruling classes impose ideologies on society that reflect their own interests, ensuring cultural hegemony in the service of capitalist accumulation, and keeping less powerful classes quiescent.

[4] Dain argues that funding is underdeveloped as a factor in our analysis, overlooking the fact that it is a central influence in the published conceptual framework and analyses from which the four challenges derive (see references numbers 25 and 41).

[5] These are not the only kinds of non-state actors that may hold power in the global governance system. Others include multinational corporations, private philanthropies, terrorist networks and religious institutions.

[6] Not all constructivists emphasize non-state power. Alexander Wendt’s *Social Theory of International Politics* (reference number 8) for instance, is a state-oriented constructivist account of power in the international system.

[7] A stronger and more defensible perspective on the role of the state in global health governance that avoids the first two of these difficulties is articulated in Robert Marten (2018): How states exerted power to create the Millennium Development Goals and how this shaped the global health agenda: Lessons for the sustainable development goals and the future of global health, Global Public Health, doi:10.1080/17441692.2018.1468474.


## References

[1] Shiffman J (2017). Four challenges that global health networks face. Int J Health Policy Manag.

[2] Dain K (2017). Challenges facing global health networks: the NCD Alliance experience: Comment on “Four challenges that global health networks face. ” Int J Health Policy Manag.

[3] Marten R, Smith RD (2017). State support: a prerequisite for global health network effectiveness: Comment on “Four challenges that global health networks face. ” Int J Health Policy Manag.

[4] Quissell K (2017). Additional insights into problem definition and positioning from social science: Comment on “Four challenges that global health networks face. ” Int J Health Policy Manag.

[5] Tosun J (2017). Polycentrism in global health governance scholarship: Comment on “Four challenges that global health networks face. ” Int J Health Policy Manag.

[6] White J (2017). The Magic pudding: Comment on “Four challenges that global health networks face. ” Int J Health Policy Manag.

[7] Wendt A (1987). The agent-structure problem in international relations theory. Int Organ.

[8] Wendt A. Social Theory of International Politics. Cambridge, UK: Cambridge University Press; 1999.

[9] Meyer JW, Boli J, Thomas GM, Ramirez FO (1997). World Society and the Nation‐State. Am J Sociol.

[10] Finnemore M, Sikkink K (1998). International Norm Dynamics and Political Change. Int Organ.

[11] Sikkink K. “Beyond the Justice Cascade: How Agentic Constructivism could help explain change in international politics.” Keynote address at Millennium Annual Conference, October 22, 2011, “Out of the Ivory Tower: Weaving the Theories and Practice of International Relations,” London, United Kingdom. https://www.princeton.edu/politics/about/file-repository/public/Agentic-Constructivism-paper-sent-to-the-Princeton-IR-Colloquium.pdf.

[12] Giddens A. The Constitution of Society: Outline of the Theory of Structuration. Berkeley, CA and Los Angeles, CA, USA: University of California Press; 1984.

[13] Foucault M. Power/Knowledge: Selected Interviews and Other Writings, 1972-1977. Colin Gordon, ed. New York: Pantheon Books; 1980.

[14] Bourdieu P. Distinction: A Social Critique of the Judgement of Taste. Cambridge, MA: Harvard University Press; 1984.

[15] Sewell WH Jr (1992). A Theory of Structure: Duality, Agency, and Transformation. Am J Sociol.

[16] Kim HJ, Sharman JC (2014). Accounts and Accountability: Corruption, Human Rights, and Individual Accountability Norms. Int Organ.

[17] Schmitz HP (2016). The global health network on alcohol control: successes and limits of evidence-based advocacy. Health Policy Plan.

[18] Gneiting U (2016). From global agenda-setting to domestic implementation: successes and challenges of the global health network on tobacco control. Health Policy Plan.

[19] Quissell K, Walt G (2016). The challenge of sustaining effectiveness over time: the case of the global network to stop tuberculosis. Health Policy Plan.

[20] Berlan D (2016). Pneumonia’s second wind? A case study of the global health network for childhood pneumonia. Health Policy Plan.

[21] Smith SL, Rodriguez MA (2016). Agenda setting for maternal survival: the power of global health networks and norms. Health Policy Plan.

[22] Shiffman J (2016). Network advocacy and the emergence of global attention to newborn survival. Health Policy Plan.

[23] Shawar YR, Shiffman J, Spiegel DA (2015). Generation of political priority for global surgery: a qualitative policy analysis. Lancet Glob Health.

[24] Shawar YR, Shiffman J (2017). Generation of global political priority for early childhood development: the challenges of framing and governance. Lancet.

[25] Shiffman J, Schmitz HP, Berlan D (2016). The emergence and effectiveness of global health networks: findings and future research. Health Policy Plan.

[26] Beach D, Pedersen RB. Process-tracing Methods: Foundations and Guidelines. Ann Arbor: University of Michigan Press; 2013.

[27] Bourdieu P. The forms of capital. In: Richardson J, ed. Handbook of Theory and Research for the Sociology of Education. New York: Greenwood; 1986:241-258.

[28] Fligstein N, McAdam D. A Theory of Fields. Oxford and New York: Oxford University Press; 2012.

[29] Mearsheimer JJ (1994). The false promise of international institutions. Int Secur.

[30] Keohane RO. After Hegemony: Cooperation and Discord in the World Political Economy. Princeton: Princeton University Press; 2005.

[31] Cox RW, Sinclair TJ. Approaches to World Order. Cambridge: Cambridge University Press; 1996.

[32] Katzenstein PJ. The Culture of National Security: Norms and Identity in World Politics. New York: Columbia University Press; 1996.

[33] Haas PM (1992). Introduction: Epistemic Communities and International Policy Coordination. Int Organ.

[34] March JG, Olsen JP. The Logic of Appropriateness. In: Gorden RE, ed. Handbook of Political Science. Oxford, UK: Oxford University Press; 2011.

[35] Sil R, Katzenstein PJ. Beyond Paradigms: Analytic Eclecticism in the Study of World Politics. London: Palgrave Macmillan; 2010.

[36] Sell SK, Prakash A (2004). Using Ideas Strategically: The Contest Between Business and NGO Networks in Intellectual Property Rights. Int Stud Q.

[37] Mitchell GE, Schmitz HP (2014). Principled instrumentalism: A theory of transnational NGO behaviour. Rev Int Stud.

[38] Robinson RS (2015). Population Policy in Sub-Saharan Africa: A Case of Both Normative and Coercive Ties to the World Polity. Popul Res Policy Rev.

[39] Keck ME, Sikkink K. Activists Beyond Borders: Advocacy Networks in International Politics. Ithaca, NY: Cornell University Press; 1998.

[40] Stop TB Partnership. http://www.stoptb.org/about/. Accessed April 24, 2018.

[41] Shiffman J, Quissell K, Schmitz HP (2016). A framework on the emergence and effectiveness of global health networks. Health Policy Plan.

[42] Ostrom E (2010). Polycentric systems for coping with collective action and global environmental change. Glob Environ Change.

[43] Shiffman J, Kunnuji M, Shawar YR, Robinson RS (2018). International norms and the politics of sexuality education in Nigeria. Global Health.

